# The Prevalence and Determinants of Undiagnosed and Diagnosed Type 2 Diabetes in Middle-Aged Irish Adults

**DOI:** 10.1371/journal.pone.0080504

**Published:** 2013-11-25

**Authors:** Jennifer M. O Connor, Seán R. Millar, Claire M. Buckley, Patricia M. Kearney, Ivan J. Perry

**Affiliations:** Department of Epidemiology & Public Health, University College Cork, Cork, Ireland; McGill University, Canada

## Abstract

**Background:**

The prevalence of type 2 diabetes within the Republic of Ireland is poorly defined, although a recent report suggested 135,000 cases in adults aged 45+, with approximately one-third of these undiagnosed. This study aims to assess the prevalence of undiagnosed and diagnosed diabetes in middle-aged adults, and compare features related to either condition, in order to investigate why certain individuals remain undetected.

**Methods:**

This was a cross-sectional study involving a sample of 2,047 men and women, aged between 50–69 years, randomly selected from a large primary care centre. Univariate logistic regression was used to explore socio-economic, metabolic and other health related variable associations with undiagnosed or diagnosed diabetes. A final multivariate analysis was used to determine odds ratios and 95% confidence intervals for having undiagnosed compared to diagnosed diabetes, adjusted for gender, age and significant covariates determined from univariate models.

**Principle Findings:**

The total prevalence of diabetes was 8.5% (95% CI: 7.4%–8.8%); 72 subjects (3.5%) had undiagnosed diabetes (95% CI: 2.8%–4.4%) and 102 subjects (5.0%) had diagnosed diabetes (95% CI: 4.1%–6.0%). Obesity, dyslipidaemia, and family history of diabetes were positively associated with both undiagnosed and diagnosed type 2 diabetes. Compared with diagnosed subjects, study participants with undiagnosed diabetes were significantly more likely to have low levels of physical activity and were less likely to be on treatment for diabetes-related conditions or to have private medical insurance.

**Conclusions:**

The prevalence of diabetes within the Cork and Kerry Diabetes and Heart Disease Study is comparable to recent estimates from the Slán National Health and Lifestyle Survey, a study which was nationally representative of the general population. A considerable proportion of diabetes cases were undiagnosed (41%), emphasising the need for more effective detection strategies and equitable access to primary healthcare.

## Introduction

Type 2 Diabetes mellitus (T2DM), a chronic disease which causes significant morbidity and mortality, was the ninth leading cause of death worldwide in 2008 [1]. Diabetes is associated with obesity, dyslipidaemia and hypertension, and is characterised by chronic hyperglycaemia due to insufficient insulin release, impaired insulin action, or a combination of both [2]. Importantly, the persistent hyperglycaemia that is associated with diabetes may cause serious health complications such as cardiovascular disease (CVD) and impairment and malfunction of the renal, ophthalmic, vascular and nervous systems. These complications pose significant financial burdens on healthcare services; research conducted in 2006, which examined economic consequences related to T2DM, estimated that almost 10% of total health expenditure was spent on diabetes care in the Republic of Ireland (ROI) alone [3].

The prevalence of T2DM is increasing globally, representing a key public health issue [4]. There is a lack of research relating to diabetes in Ireland, although recent studies have indicated that the condition may be reaching epidemic proportions [5], [6]. In 1998, the prevalence of T2DM amongst subjects in a primary care based sample was estimated to be 3.9% [7]. A recent report from the Irish Institute of Public Health (IPH) [8] based on findings from the 2007 Slán National Health and Lifestyle Survey [9], suggested a prevalence of 8.9% in adults aged 45+. This estimate consisted of 94,000 subjects who had clinically diagnosed T2DM and 41,000 with undiagnosed diabetes. While the efficacy and cost-effectiveness of routine screening for diabetes in primary care has not been established [10]–[12], there is an ongoing need for contemporary data on the prevalence of T2DM, in population and primary care settings, in order to guide policy in this area. This could help formulate strategies that further develop effective diabetes prevention, detection and management, as individuals with undiagnosed T2DM are at high risk of diabetic complications [13].

The aim of this study was to estimate the prevalence of both undiagnosed and diagnosed T2DM in a sample of men and women aged 50–69 years, drawn from a primary care setting similar to that studied in 1998 [7], using the same field survey procedures and methods. In particular, we determined the extent to which the probability of T2DM diagnosis is influenced by access to primary care as defined by health insurance status.

## Methods

### Study population

This research makes use of data from the Cork and Kerry Diabetes and Heart Disease Study (Phase II), a single centre, cross-sectional study conducted between 2010 and 2011. A population representative random sample was recruited from a large primary care centre in Mitchelstown, County Cork, Ireland. The Livinghealth Clinic includes eight General Practitioners (GP) and serves a population of approximately 20,000, with a mix of urban and rural residents. The name, address, gender and date of birth were provided for all registered attending patients and stratified random sampling by age and sex was employed to recruit equal numbers of men and women in four quartiles between the ages of 50 and 69 years. In total, 3,807 potential participants were selected from the practice list. Following the exclusion of duplicates, deaths, and subjects incapable of consenting or attending appointment, 3,051 were invited to participate in the study and of these, 2,047 (49.2% male) completed the questionnaire and physical examination components of the baseline assessment (response rate: 67.1%). The status of non-responders included individuals refusing to participate (59.4%) and those who did not reply (40.6%). Male subjects accounted for 53.7% of non-responders while 43.5% (vs. 42.8% of responders) were >60 years of age. All non-responders were followed up with a phone call where possible and otherwise with a single postal reminder. Details regarding the study design, sampling procedures and methods of data collection have been reported previously [14].

Ethics committee approval conforming to the Declaration of Helsinki was obtained from the Clinical Research Ethics Committee of University College Cork. A letter signed by the contact GP in the clinic was sent out to all selected participants with a reply slip indicating acceptance or refusal. All subjects gave signed informed consent, including permission to use their data for research purposes.

### Clinical and laboratory measurements

The weight and height of each subject were measured to the nearest 0.1 kg and 0.1 cm respectively by trained researchers. Study participants were asked to remove heavy outer clothing and footwear. Portable electronic Tanita WB-100MA weighing scales (Tanita Corporation, IL, USA) were placed on a firm, flat surface and were calibrated weekly to ensure accuracy. Height was measured using a portable Seca Leicester height/length stadiometer (Seca, Birmingham, UK). Two measurements of weight and height were taken for each subject and the mean value of these were used in the analysis. Three measurements of systolic and diastolic blood pressure (SBP and DBP respectively) were obtained with the subject in a seated position using an Omron M7 digital sphygmomanometer (Omron Healthcare Co. Ltd., Japan). The mean of the second and third readings was considered as a subject's blood pressure. After an overnight fast, all participants were invited to attend the clinic for the sampling of blood between 8 and 10 A.M. Triglyceride (TAG), high density lipoprotein cholesterol (HDL-C) and fasting plasma glucose (FPG) levels were measured by Cork University Hospital Biochemistry Laboratory on Olympus biochemistry analysers with Olympus reagents using standardised procedures and fresh samples (Olympus Diagnostica Gmbh, Hamburg, Germany). Glucose concentrations were determined using a glucose hexokinase assay (Olympus Life and Material Science Europa Ltd., Lismeehan, Co. Clare, Ireland). Glycated haemoglobin A_1c_ (HbA_1c_) levels were measured in the haematology laboratory on an automated high-pressure liquid chromatography instrument Tosoh G7 (Tosoh HLD-723 (G7), Tosoh Europe N.V, Tessenderlo, Belgium). A self-administered General Health Questionnaire (GHQ) was used to collect supplementary information which included medication use, demographic characteristics, medical cover, family T2DM history, past medical history of CVD and smoking and alcohol behaviours. Physical activity levels were assessed using the validated International Physical Activity Questionnaire (IPAQ) [15].

### Metabolic and anthropometric classifications

Metabolic features were categorised according to International Diabetes Federation metabolic syndrome (MetS) criteria cut-points [16]. Abnormal metabolic risks were defined as FPG ≥5.6 mmol/L, TAG ≥1.7 mmol/L and HDL-C <1.03 mmol/L in males and HDL-C <1.29 mmol/L in females. Dyslipidaemia was determined according to elevated TAG and low HDL-C levels. Hypertension was defined as SBP ≥140 mmHg) and/or DBP ≥90 mmHg [17]. Body Mass Index (BMI) was calculated by dividing a subject's weight by the square of their height and was categorised as <25 =  *Normal-weight*, 25–29.9 =  *Overweight*, and ≥30 =  *Obese* [18], [19].

### Morbidity

Type 2 diabetes was defined as HbA_1c_ ≥6.5% (N = 146). Undiagnosed diabetes was determined if subjects had positive HbA_1c_ tests but did not report a medical diagnosis of T2DM or oral medication use for the condition (N = 72). Diagnosed diabetes was classified according to positive test results and self-reported doctor diagnosis or diabetes medication use (N = 74), or by diagnosis or medication use alone (N = 28, total diagnosed = 102). The presence of CVD was obtained from the GHQ by asking study participants if they had been diagnosed with one of the following seven conditions: *Heart Attack* (including coronary thrombosis or myocardial infarction), *Heart Failure*, *Angina*, *Aortic Aneurysm, Hardening of the Arteries*, *Stroke* or any other *Heart Trouble*. Subjects indicating a diagnosis of any of these disorders were classified as having CVD.

### Covariates

Covariates utilised from the GHQ included gender, age, use of prescription (Rx) anti-hypertensive and cholesterol-lowering medication, family T2DM or CVD history, education, social class, medical cover, physical activity levels, smoking status and alcohol use. Age was included either as a dichotomous (<60/≥60 years of age) or continuous variable in univariate or multivariate regression models. Education was divided into four categories: *Primary, Secondary, Diploma* and *Bachelor or Higher*. Social class was defined according to the European Socio-economic Classification System (ESeC) [20], [21], and collapsed into three groups: *High Income, Middle Income and Low Income*. The health service variables – *Private Insurance, No Insurance*, and means-tested, state-assisted *General Practice Visit Card* (GPC) and *Full Medical Card* (FMC) – were transformed into a dummy variable: *Private Insurance, State Insurance, No Insurance*. Subjects reporting more than one insurance type were assigned to the higher insurance category. Self-reported physical activity within the previous six months, measured using the IPAQ questionnaire [15], was divided into three categories: *High, Moderate* and *No Physical Exercise*. Alcohol use was assessed by asking study participants how often they consumed alcohol on a monthly or weekly basis, and was classified as follows: ‘never or less than once a month’ - *Non-drinker*, ‘2–4 times monthly’ - *Occasional Drinker*, and ‘twice or more weekly’ - *Regular Drinker*. Subjects were considered current smokers if they smoked cigarettes during the recruitment phase, had smoked within the last 10 years or had smoked more than 100 cigarettes in their lifetime, and non-smokers if they had smoked less than this or had never smoked.

### Statistical analysis

The descriptive statistics (mean, median, standard deviation, inter-quartiles and percentage distribution) of the study population were examined by diabetes status. Gender differences in T2DM prevalence were compared using chi-square tests. The relationships between health conditions, health behaviours, health insurance status and metabolic/socio-economic factors for individuals with undiagnosed or diagnosed T2DM were explored through multiple univariate binary logistic regressions. Diagnosed subjects were excluded from models examining undiagnosed T2DM, while models investigating associations between features and diagnosed diabetes excluded undiagnosed cases. Distinctions between undiagnosed and diagnosed T2DM were explored in univariate analyses excluding non-diabetic participants.

To further compare feature/morbidity relationships and strengths of association to either undiagnosed or diagnosed T2DM, multivariate logistic regressions were performed. To select independent predictor variables (IPV) to be included in analysis, IPVs that had a P-value of less than 0.2 in univariate models were included in stepwise forward and backwards entry elimination multivariate analysis, with model stability assessed using the likelihood ratio (LR). Variables indicating a significant relationship (P<0.05) with either condition were then entered sequentially, by order of magnitude of the chi-square association, into two independent logistic regressions, adjusted for gender and age as a dichotomous (<60/≥60) variable. Using the same procedures, a final multivariate model comparing undiagnosed to diagnosed T2DM was determined, adjusted for gender and age as a continuous measure.

The discriminatory properties of specific IPVs indentified in multivariate analysis were evaluated. Models including these variables were assessed for their ability to detect undiagnosed or diagnosed T2DM using the *c* statistic. The *c* statistic is identical to the area under the receiver operating characteristic curve (AUC) with values ranging from 0.5 (no better than chance) to 1.0 (indicating perfect discrimination) [22].

Primary data analysis was conducted using PASW Statistics version 18 (SPSS, Chicago, IL, USA) for Windows. Confidence intervals for prevalence proportions were calculated using the VasserStats statistical website [23]. For all analyses, a P-value (two-tailed) of less than 0.05 was considered to indicate statistical significance. Glycated haemoglobin A_1c_ test results and diagnostic status information were available for 1,995 (97.5%) and 1,999 (97.7%) subjects respectively. Missing dichotomous predictor data values were assumed to be negative, while missing continuous data were assumed to be ignorable and missing at random.

## Results

The baseline characteristics of the study population for participants with undiagnosed, diagnosed and no diabetes are shown in table 1. The total prevalence of T2DM was 8.5% (95% CI: 7.4%–8.8%); 102 (5.0%) subjects had diagnosed diabetes (95% CI: 4.1%–6.0%) and 72 (3.5%) had undiagnosed diabetes (95% CI: 2.8%–4.4%), representing 41.4% of all diabetes cases. A significantly greater proportion of male subjects 11.1% (N = 112) had T2DM compared to females 6.0% (N = 62, P<0.001), and a greater proportion of males had both undiagnosed and diagnosed T2DM. A high proportion of diabetes cases were overweight or obese, used Rx anti-hypertensive and cholesterol-lowering medications, had a family history of diabetes or previous history of CVD, finished education at primary level and reported having low levels of physical activity within the previous six months. Variations in health insurance were also noted, with a greater proportion of T2DM subjects having state-assisted healthcare.

**Table 1:** Characteristics of the study population.^1^

Feature	No diabetes	Undiagnosed diabetes	Diagnosed diabetes
	N = 1873 (91.5%)	N = 72 (3.5%)	N = 102 (5.0%)
***Health conditions***			
Male	893 (47.8)	43 (59.7)	69 (67.6)
Age	59.0 (54.0–64.0)	60.0 (56.3–65.0)	62.0 (57.0–65.0)
Age ≥60	875 (46.9)	38 (52.8)	65 (63.7)
On Rx for hypertension	486 (26.0)	32 (44.4)	66 (64.7)
On Rx for cholesterol	609 (32.6)	35 (48.6)	67 (65.7)
BMI (kg/m^2^)	28.29±4.6	33.06±6.3	31.19±4.4
** BMI category:**			
* <25*	439 (23.6)	4 (5.6)	2 (2.0)
* 25*–*29.9*	857 (46.0)	24 (33.3)	43 (42.2)
≥*30*	566 (30.4)	44 (61.1)	57 (55.9)
Family history of T2DM	315 (16.9)	21 (29.2)	54 (52.9)
CVD	167 (8.9)	16 (22.2)	29 (28.4)
***Socio-economic***			
** Education:**			
* Bachelor or higher*	175 (10.0)	4 (5.9)	5 (5.3)
* Diploma*	239 (13.7)	6 (8.8)	6 (6.3)
* Secondary*	863 (49.5)	31 (45.6)	40 (42.1)
* Primary only*	466 (26.7)	27 (39.7)	44 (46.3)
** Social class:**			
* High income*	244 (18.2)	6 (11.5)	11 (13.3)
* Middle income*	396 (29.5)	18 (34.6)	25 (30.1)
* Low income*	704 (52.4)	28 (53.8)	47 (56.6)
***Medical cover***			
** Health insurance:**			
* Private insurance*	1196 (64.0)	27 (37.5)	51 (50.0)
* State insurance*	437 (23.4)	29 (40.3)	44 (43.1)
* No insurance*	236 (12.6)	16 (22.2)	7 (6.9)
***Health behaviours***			
** Physical activity:**			
* High*	795 (48.4)	10 (17.5)	31 (34.8)
* Moderate*	536 (32.6)	19 (33.3)	35 (39.3)
* No physical exercise*	313 (19.0)	28 (49.1)	23 (25.8)
Smoker	889 (47.6)	38 (52.8)	60 (58.8)
** Alcohol use:**			
* Non-drinker*	800 (44.7)	38 (55.1)	54 (53.5)
* Occasional drinker*	367 (20.5)	12 (17.4)	27 (26.7)
* Regular drinker*	623 (34.8)	19 (27.5)	20 (19.8)
***Metabolic***			
FPG (mmol/L)	4.90 (4.6–5.3)	6.60 (5.6–7.5)	7.50 (5.7–9.4)
FPG ≥5.6	238 (13.1)	58 (80.6)	80 (80.8)
TAG (mmol/L)	1.19 (0.9–1.6)	1.80 (1.3–2.4)	1.36 (1.0–2.0)
TAG≥1.7	417 (23.0)	37 (52.9)	36 (37.5)
HDL-C (mmol/L)	1.48±0.4	1.22±0.3	1.18±0.3
HDL-C (non-optimal)^2^	267 (14.7)	32 (45.7)	45 (45.0)
Dyslipidaemia^3^	122 (6.7)	24 (34.3)	21 (21.0)
SBP (mmHg)	129.25±16.7	134.18±19.3	132.94±16.4
DBP (mmHg)	80.20±9.7	80.12±10.9	78.79±9.5
Hypertension^4^	552 (29.7)	28 (39.4)	28 (27.5)

1 Mean and ± SD are shown for continuous and % are shown for categorical variables. Age, FPG and TAG are shown as a median (interquartile range). Numbers and % (in brackets) for categorical variables will vary in different analyses as some variables have missing values.

2 HDL-C: <1.03 (MALES) <1.29 (FEMALES).

3 Dyslipidaemia: TAG≥1.7 and HDL-C: <1.03 (MALES) <1.29 (FEMALES).

4 Hypertension: SBP≥140 and/or DBP≥90.

In univariate analysis (table S1), overweight and obesity, family diabetes and CVD history, elevated TAG, low HDL-C and dyslipidaemia were significantly associated with both undiagnosed and diagnosed T2DM. Associations between reduced physical activity levels and T2DM were noticeably strong, with seven-fold and approximate two-fold increased odds for undiagnosed or diagnosed T2DM respectively. With regard to health services related factors, there was a two-fold increased likelihood of undiagnosed T2DM in patients on treatment for hypertension versus a five-fold increased odds for diagnosed diabetes. Similarly, the odds of having undiagnosed T2DM were approximately two-fold higher in patients on treatment with cholesterol-lowering therapy versus an approximate four-fold increase for diagnosed diabetes. The probability of both undiagnosed and diagnosed T2DM was significantly reduced in patients with private medical insurance, whilst the odds of having undiagnosed diabetes were significantly increased in subjects with no medical insurance (OR: 3.0, 95% CI: 1.6–5.6).

Multivariate analysis (table 2) revealed overweight and obesity, use of cholesterol-lowering medication, family T2DM history and dyslipidaemia to be associated with both undiagnosed and diagnosed T2DM. Low level physical activity (OR: 5.8, 95% CI: 2.7–12.5) and health service variables remained significant determinants of undiagnosed diabetes, with odds that were approximately two-fold higher in subjects with state-assisted healthcare and for participants without medical insurance. Characteristics associated with diagnosed T2DM included CVD history, Rx anti-hypertensive therapy and alcohol use. In addition, male subjects were statistically more likely to have diagnosed diabetes compared to females (OR: 2.5, 95% CI: 1.5–4.1).

**Table 2:** Odds ratios (95% CI) of having undiagnosed or diagnosed type 2 diabetes compared to no diabetes – multivariate logistic regression adjusted for gender, age and all significant covariates.

Feature	Odds ratio	95% CI
***Undiagnosed T2DM compared to no diabetes*** 1		
Male	1.4	(0.8–2.5)
Age≥60	1.0	(0.6–1.9)
On Rx for cholesterol	2.2	(1.2–3.9)
** BMI category:**		
* <25*	1	
* 25-29.9*	4.5	(1.0–19.5)
≥*30*	6.8	(1.6–29.4)
Family history of T2DM	1.9	(1.0–3.6)
** Health insurance:**		
* Private insurance*	1	
* State insurance*	2.2	(1.2–4.2)
* No insurance*	2.3	(1.0–5.2)
** Physical activity:**		
* High*	1	
* Moderate*	1.9	(0.8–4.2)
* No physical exercise*	5.8	(2.7–12.5)
Dyslipidaemia^3^	4.3	(2.3–8.3)
***Diagnosed T2DM compared to no diabetes*** 2		
Male	2.5	(1.5–4.1)
Age≥60	1.4	(0.9–2.3)
On Rx for hypertension	2.7	(1.7–4.4)
On Rx for cholesterol	2.0	(1.2–3.3)
** BMI category:**		
* <25*	1	
* 25*–*29.9*	8.2	(1.9–34.6)
≥*30*	9.4	(2.2–40.3)
Family history of T2DM	5.9	(3.7–9.4)
CVD	2.0	(1.1–3.5)
** Alcohol use:**		
* Non-drinker*	1	
* Occasional drinker*	1.3	(0.7–2.2)
* Regular drinker*	0.4	(0.2–0.7)
Dyslipidaemia^3^	1.9	(1.0–3.5)

1 Model excludes subjects with diagnosed T2DM. Final model covariates entered in order: dyslipidaemia, BMI

category, physical activity, health insurance, on Rx for cholesterol, family history of T2DM, gender and age.

2 Model excludes subjects with undiagnosed T2DM. Final model covariates entered in order: family history of T2DM,

on Rx for hypertension, BMI category, on Rx for cholesterol, CVD, dyslipidaemia, alcohol use, gender and age.

3 Dyslipidaemia: TAG≥1.7 and HDL-C: <1.03 (MALES) <1.29 (FEMALES).

Table 3 shows univariate odds ratios for undiagnosed compared to diagnosed T2DM. Within this sub-sample of diabetes cases, significant effects were observed for medication use, family T2DM history, TAG levels and dylipidaemia. Both health insurance and physical activity IPVs demonstrated strong associations for having undiagnosed T2DM, with approximate four-fold increased odds in subjects without healthcare insurance and in those reporting low levels of physical activity. Individuals with undiagnosed T2DM were also more likely to have a higher BMI. Overall, metabolic features were less optimal in undiagnosed cases, and a greater proportion had uncontrolled hypertension.

**Table 3:** Univariate odds ratios (95% CI) of having undiagnosed compared to diagnosed type 2 diabetes.^1^

Feature	Undiagnosed diabetes	Diagnosed diabetes	Odds ratio	95% CI
	N = 72 (41.4%)	N = 102 (58.6%)		
***Health conditions***				
Female	29 (40.3)	33 (32.4)	1	
Male	43 (59.7)	69 (67.6)	0.7	(0.4–1.3)
Age <60 years	34 (47.2)	37 (36.3)	1	
Age≥60 years	38 (52.8)	65 (63.7)	0.6	(0.3–1.2)
Not on Rx for hypertension	40 (55.6)	36 (35.3)	1	
On Rx for hypertension	32 (44.4)	66 (64.7)	0.4	(0.2–0.8)
Not on Rx for cholesterol	37 (51.4)	35 (34.3)	1	
On Rx for cholesterol	35 (48.6)	67 (65.7)	0.5	(0.3–0.9)
BMI (kg/m^2^)	33.06±6.3	31.19±4.4	1.1	(1.0–1.1)
** BMI category:**				
* <25*	4 (5.6)	2 (2.0)	1	
* 25*–*29.9*	24 (33.3)	43 (42.2)	0.3	(0.1–1.7)
≥*30*	44 (61.1)	57 (55.9)	0.4	(0.1–2.2)
No family history of T2DM	51 (70.8)	48 (47.1)	1	
Family history of T2DM	21 (29.2)	54 (52.9)	0.4	(0.2–0.7)
No CVD	56 (77.8	73 (71.6)	1	
CVD	16 (22.2)	29 (28.4)	0.7	(0.4–1.5)
***Socio-economic***				
** Education:**				
* Bachelor or higher*	4 (5.9)	5 (5.3)	1	
* Diploma*	6 (8.8)	6 (6.3)	1.3	(0.2–7.1)
* Secondary*	31 (45.6)	40 (42.1)	1.0	(0.2–3.9)
* Primary only*	27 (39.7)	44 (46.3)	0.8	(0.2–3.1)
** Social class:**				
* High income*	6 (11.5)	11 (13.3)	1	
* Middle income*	18 (34.6)	25 (30.1)	1.3	(0.4–4.2)
* Low income*	28 (53.8)	47 (56.6)	1.1	(0.4–3.3)
***Medical cover***				
Health insurance:				
* Private insurance*	27 (37.5)	51 (50.0)	1	
* State insurance*	29 (40.3)	44 (43.1)	1.2	(0.6–2.4)
* No insurance*	16 (22.2)	7 (6.9)	4.3	(1.6–11.8)
***Health behaviours***				
** Physical activity:**				
* High*	10 (17.5)	31 (34.8)	1	
* Moderate*	19 (33.3)	35 (39.3)	1.7	(0.7–4.2)
* No physical exercise*	28 (49.1)	23 (25.8)	3.8	(1.5–9.3)
Non-smoker	34 (47.2)	42 (41.2)	1	
Smoker	38 (52.8)	60 (58.8)	0.8	(0.4–1.4)
** Alcohol use:**				
* Non-drinker*	38 (55.1)	54 (53.5)	1	
* Occasional drinker*	12 (17.4)	27 (26.7)	0.6	(0.3–1.4)
* Regular drinker*	19 (27.5)	20 (19.8)	1.4	(0.6–2.9)
***Metabolic***				
TAG (mmol/L)	1.80 (1.3–2.4)	1.36 (1.0–2.0)	1.5	(1.1–2.0)
TAG <1.7	33 (47.1)	60 (62.5)	1	
TAG≥1.7	37 (52.9)	36 (37.5)	1.9	(1.0–3.5)
HDL-C (mmol/L)	1.22±0.3	1.18±0.3	1.7	(0.6–4.7)
Optimal HDL-C	38 (54.3)	55 (55.0)	1	
Non-optimal HDL-C^2^	32 (45.7)	45 (45.0)	1.0	(0.6–1.9)
No dyslipidaemia	46 (65.7)	79 (79.0)	1	
Dyslipidaemia^3^	24 (34.3)	21 (21.0)	2.0	(1.0–3.9)
SBP (mmHg)	134.18±19.3	132.94±16.4	1.0	(0.99–1.0)
DBP (mmHg)	80.12±10.9	78.79±9.5	1.0	(0.98–1.0)
No hypertension	43 (60.6)	74 (72.5)	1	
Hypertension^4^	28 (39.4)	28 (27.5)	1.7	(0.9–3.3)

1 Mean and ± SD are shown for continuous variables. TAG is shown as a median (interquartile range). Numbers and % (in brackets) for categorical variables will vary in different analyses as some variables have missing values.

2 HDL-C: <1.03 (MALES) <1.29 (FEMALES).

3 Dyslipidaemia: TAG≥1.7 and HDL-C: <1.03 (MALES) <1.29 (FEMALES).

4 Hypertension: SBP≥140 and/or DBP≥90.

Results from multivariate analysis comparing undiagnosed to diagnosed T2DM are presented in table 4. Significant associations were noted for BMI (continuous) and physical inactivity. Undiagnosed participants were significantly less likely to be on treatment for hypertension or to have a family history of T2DM relative to subjects with diagnosed diabetes.

**Table 4:** Odds ratios (95% CI) of having undiagnosed compared to diagnosed type 2 diabetes – multivariate logistic regression adjusted for all significant covariates.^1^

Feature	Model 1		Model 2^2^	
	Odds ratio	95% CI	Odds ratio	95% CI
BMI (kg/m^2^)	1.1	(1.0–1.2)	1.1	(1.0–1.2)
On Rx for hypertension	0.3	(0.2–0.7)	0.3	(0.1–0.7)
Family history of T2DM	0.4	(0.2–0.8)	0.4	(0.2–0.8)
Physical activity:				
*High*	1		1	
*Moderate*	1.6	(0.6–4.3)	1.6	(0.6–4.3)
*No physical exercise*	3.5	(1.3–9.3)	3.4	(1.3–9.1)

1 Final model covariates entered in order: family history of T2DM, physical activity, on Rx for hypertension and BMI.

2 Adjusted for gender and age.

Figures S1 and S2 show AUCs for models to discriminate undiagnosed or diagnosed T2DM (compared to no diabetes). Models which included both health insurance and physical activity IPVs showed a higher discriminatory capacity to detect undiagnosed T2DM (*c*: 0.735, 95% CI: 0.668–0.801) compared to diagnosed T2DM (*c*: 0.608, 95% CI: 0.544–0.671). A model including health insurance, physical activity and BMI (continuous) displayed further improved discrimination, (*c*: 0.814, 95% CI: 0.758–0.871) for undiagnosed T2DM vs. (*c*: 0.698, 95% CI: 0.646–0.750) for diagnosed subjects.

## Discussion

The results from previous research investigating the prevalence of T2DM within the ROI are conflicting. In 1998 a study conducted by Perry et al. [7] suggested an overall prevalence of 3.9%, 30% of whom were undiagnosed, whereas research in 2003, examining T2DM in primary care [24], estimated a population prevalence of 9.2%, with undiagnosed subjects representing 23.5% of all cases. The variance between these studies is possibly explained by the differences in age groups assessed, or by methods used for diabetes detection. The higher prevalence of undiagnosed T2DM identified within the present study population may be due to use of the HbA_1c_ procedure as compared to the FPG test that was more commonly employed in the ROI before 2010. Research conducted in Germany and the United States (US), which compared FPG and Oral Glucose Tolerance Test methods, reported that overall prevalence of T2DM would have been lower had diabetes been classified by FPG [25], [26]. The present study also observed that 14 (19%) undiagnosed subjects (who were positively identified according to HbA_1c_ concentrations) had FPG levels that were less than 5.6 mmol/L, and would have been classified as non-diabetic if this method had been used for diagnostic purposes within the Cork and Kerry Study. This finding is consistent with other studies which have reported variations between HbA_1c_ and FPG [27]–[29]. Although a recent report from the US implied that use of the HbA_1c_ assay would not significantly alter T2DM prevalence and that diabetes categorisation would remain unchanged in 97.7% of subjects [30], evidence is still equivocal [31]. Several studies have shown poor concordance between HbA_1c_ and FPG [27], in particular regarding pre-diabetes classification [32]–[34]. Additionally, factors such as age or ethnicity are thought to influence results [27], [28], [35]. Nevertheless, as discussed by Bonora et al., comparisons between diagnostic methods for T2DM detection are ambiguous, as a true gold standard test is unavailable [36].

The recent Irish IPH report [8], based on the nationally representative 2007 Slán National Health and Lifestyle Survey [9] (which also used the HbA_1c_ test), estimated the prevalence of T2DM in adults 45+ to be 8.9%, which is similar to the result suggested by this study. In the IPH report, undiagnosed diabetes prevalence was determined to be 2.7% (30% of all diabetes cases). Of note, however, is that the IPH research estimated the prevalence of both undiagnosed and diagnosed T2DM in adults aged between 55–64 to be 4.6% and 6.3% respectively, which are comparable to outcomes attained from this study population (3.6% and 5.6%), for the same age group. Also of interest, is that results from the Slán data are consistent with the present study's finding that the prevalence of T2DM in middle-aged subjects within the ROI is higher in men. Although this gender disparity may be a consequence of selection bias due to non-response, similarity in outcomes between the 2007 Slán survey and the Cork and Kerry Diabetes and Heart Disease Study imply that observed prevalence estimates are valid. It is possible that the lower prevalence of T2DM in women may be as a result of random opportunistic screening due to higher GP consultation rates observed in females [37]. An alternative explanation may be the higher prevalence of overweight and obesity observed in male subjects within this population (males: 85.8% vs. females: 70.6%, P for difference <0.001), a relationship noted in previous research examining obesity within Ireland [37], [38].

As numerous studies have indicated, non-optimal metabolic risk factors such as elevated TAG and low HDL-C were significantly and positively associated with both undiagnosed and diagnosed T2DM [25], [26], [39]. A noticeable feature of undiagnosed subjects was the higher percentage of cases with uncontrolled hypertension, increased TAG concentrations and dyslipidaemia, perhaps reflecting access to treatment, as a greater proportion of diagnosed subjects used Rx anti-hypertensive and cholesterol-lowering medications. Undiagnosed individuals were also less likely to have a family history of T2DM and CVD, or to engage in regular physical activity compared to diagnosed subjects. Nevertheless, unfavourable lipid profiles, T2DM history, low level physical activity and CVD were all positively associated with both undiagnosed and diagnosed diabetes. The inverse association between diagnosed T2DM and alcohol intake was also of interest as correlations between alcohol use and MetS have been reported previously [40]. Markedly, 96.6% (N = 168) of study participants with both undiagnosed and diagnosed T2DM were either overweight or obese, confirming results from previous research which suggests that obesity is a primary and significant risk factor related to diabetes development [41]. Screening for T2DM may be more efficient within these subgroups, particularly individuals with a combination of these features.

Within the ROI, residents accessing public healthcare are divided into two categories: (1) those who hold a medical card (either a FMC or GPC) and thus qualify for means-tested, state-assisted healthcare insurance. A FMC entitles individuals to free GP services, Rx medications, public hospital services, dental, optical and aural services, community care and personal social services. A GPC entitles individuals to free GP care; (2) non-card holders, who are entitled to free public hospital services but who must pay for GP care and may also have to pay in-patient and out-patient hospital charges. In addition to the public health system there is also a large private healthcare market [42]. Results from the present study suggest that within this population, subjects with private medical insurance are less likely to have undiagnosed or diagnosed diabetes. This may indicate that these individuals have greater financial resources and access to screening and healthcare, or an increased awareness of risk factors related to T2DM. This awareness could be due to higher educational levels, as it was also noted that study participants who had only completed education to a primary level were more likely to have diabetes. Although social class (defined by the ESeC) was not an associated risk factor for having either undiagnosed or diagnosed T2DM, it is possible that the lower prevalence of diabetes amongst subjects with private medical insurance was due to socio-economic inequalities, as study participants in receipt of state-assisted medical insurance were notably at a higher risk. These findings suggest that diabetes cases occur disproportionately amongst individuals who are economically deprived and have lower education levels, and this concurs with previous research which found significant correlations between social deprivation and T2DM [43].

Importantly, these results also imply that health service inequalities are significant determinants of diagnostic status, as a greater proportion of undiagnosed cases indicated having no state-assisted or private healthcare insurance. This is consistent with outcomes observed in previous studies which have examined relationships between healthcare inequities and T2DM [13], [44]. Univariate analysis suggested three-fold and four-fold increased odds of having undiagnosed T2DM in subjects without medical insurance when compared to individuals with no diabetes (table S1) or diagnosed T2DM (table 3) respectively. This association was also noted in a multivariate logistic regression comparing undiagnosed to non-diabetic individuals (table 2) but was not observed in multivariate analysis restricted to subjects with T2DM (table 4).

To investigate this discrepancy, we forced the health insurance IPV into a model and entered covariates independently to assess confounder-adjusted relationships. In a logistic regression which controlled for family T2DM history, Rx anti-hypertensives, BMI, age and gender, having no healthcare insurance remained strongly associated with undiagnosed T2DM (OR: 3.5, 95% CI: 1.2–10.4, P = 0.025) although this was attenuated when the physical activity IPV was included (OR: 2.4, 95% CI: 0.7–8.9, P = 0.184). This may indicate a relationship between physical activity and both health insurance and undiagnosed T2DM or that physical activity levels explain most of the variance. Equally possible is that missing data from the IPAQ questionnaire resulted in a loss of statistical power.

We further explored health insurance/physical activity relationships with undiagnosed/diagnosed T2DM using the LR. Tests for model assessment included significant covariates, age, gender and either health insurance or physical activity IPVs. Both models implied similar goodness of fit (LR chi-square: 33.29, P<0.001 for a model with health insurance vs. LR chi-square: 32.68, P<0.001 for a model with physical activity) in full models against a constant, indicating that both predictors may be clinically relevant. In addition, it was noted that models including health insurance and physical activity IPVs displayed variations in discriminatory ability to detect either undiagnosed or diagnosed T2DM (figures S1 and S2). This suggests that use of these variables in T2DM risk prediction scores may be useful for identifying a subset of diabetes cases.

### Strengths and limitations

As one of the largest cross-sectional studies performed to date within the ROI, the Cork and Kerry Diabetes and Heart Disease Study sample size is comparable to other related Irish studies. Selection bias was minimised as a similar number of male and female subjects, aged between 50–69 years of age, were randomly selected from a register of patients within a single primary care based sample. Furthermore, non-responders had similar numbers for both males and females and likewise for age groups. Few studies have assessed the prevalence of undiagnosed or diagnosed T2DM within one broadly representative population sample or compared features between undiagnosed and diagnosed subjects. Finally, use of the HbA_1c_ measurement provided prevalence rates comparable to those from a recent nationally representative study: the 2007 Slán National Health and Lifestyle Survey [9].

Notwithstanding these strengths, several limitations can be identified. The use of self-reported questionnaires is subject to potential inaccuracies, recall and reporting bias [45], [46]. Misclassification of diabetes from self-reporting is a recognised limitation present in all surveys, and is a particular restraint in the ROI due to the absence of a unique health identifier within the Irish healthcare system [47]. This makes linkage with other records, such as disease registries or death records problematic [14]. Nonetheless, several studies have indicated a reasonable or high degree of concordance between T2DM prevalence and self-reporting [45], [48]–[50] and whenever possible empirical methods were used in analysis. Additionally, within this sample there was a high level of agreement between self-reported doctor diagnosis of T2DM and Rx diabetes medication use (Kappa: 0.854, 95% CI: 0.796–0.912, P<0.001).

Equally of concern is that prevalence estimates were derived from a single primary care based sample which may not be representative of the general Irish population. However, previous research suggests that approximately 98% of Irish adults are registered with a GP and that, even in the absence of a universal patient registration system, it is possible to perform population based epidemiological studies that are representative of the general population using these methods [51]. Further studies are needed to definitively confirm this conclusion. If correct, it may indicate that findings from the Cork and Kerry Diabetes and Heart Disease Study are generalisable to the Irish population aged between 50–69 years. Also, as this research makes use of cross-sectional data, interpretations of these findings are compromised by the inability to infer causal relationships. Nevertheless, the relationships described have been extensively replicated in other prospective cohort studies. Finally, with regard to statistical procedures employed in analysis, the possibility of model over-fitting or type II errors cannot be discounted, and results should be considered preliminary and exploratory, as future studies with larger sample sizes and greater statistical power might find other relationships [52].

### Conclusions

The prevalence of T2DM within the ROI is consistent with trends worldwide [53], [54], and is primarily driven by the increasing obesity epidemic [4], [55]. Despite policies and continued investment in services which promote awareness and knowledge of a disease that is largely preventable, the prevalence of diabetes in Ireland is rising [8].

Socio-economic and health service inequalities are significant risk factors for having undiagnosed T2DM. The results from this study indicate that subjects with state-subsidised healthcare insurance, and those without private or state-assisted medical cover, are more likely to be undetected. These findings suggest that individuals from lower socio-economic backgrounds should be targeted. Observed low levels of physical activity, obesity level assessment and recognition of untreated cardiovascular conditions may also improve identification of T2DM cases within clinical practice. Finally, as a successful programme to detect subjects with T2DM may depend on regular General Practice attendance, a strategic approach which identifies individuals without access to primary health services and which furthers efforts to promote affordable and equitable healthcare, is needed to prevent predictable sequelae for affected individuals and populations.

## Supporting Information

Figure S1 **Area under the receiver operating characteristic curves (AUC) for models to discriminate undiagnosed type 2 diabetes compared to no diabetes.** The figure shows area under the curves for models to detect undiagnosed type 2 diabetes. The *c* statistics values were: (1) *c*: 0.735, (95% CI: 0.668–0.801) for a model including health insurance and physical activity; (2) *c*: 0.814, (95% CI: 0.758–0.871) for a model including health insurance, physical activity and BMI (continuous).
(DOCX)

Figure S2 **Area under the receiver operating characteristic curves (AUC) for models to discriminate diagnosed type 2 diabetes compared to no diabetes.** The figure shows area under the curves for models to detect diagnosed type 2 diabetes. The *c* statistics values were: (1) *c*: 0.608, (95% CI: 0.544–0.671) for a model including health insurance and physical activity; (2) *c*: 0.698, (95% CI: 0.646–0.750) for a model including health insurance, physical activity and BMI (continuous).
(DOCX)

Table S1 Univariate odds ratios (95% CI) of having undiagnosed or diagnosed type 2 diabetes compared to no diabetes. The table displays univariate associations for socio-economic, metabolic and other health related variables with either undiagnosed or diagnosed diabetes. Diagnosed subjects were excluded from models examining undiagnosed diabetes. Undiagnosed subjects were excluded from models examining diagnosed diabetes.
(DOCX)
