# Supplementary material for: The Prevalence and Determinants of Undiagnosed and Diagnosed Type 2 Diabetes in Middle-Aged Irish Adults
Source: PLoS One. 2013 Nov 25;8(11):e80504. doi: 10.1371/journal.pone.0080504 (PMC3840064; doi:10.1371/journal.pone.0080504)

**Figure S1.** Area under the receiver operating characteristic curves (AUC) for models to discriminate undiagnosed type 2 diabetes compared to no diabetes.


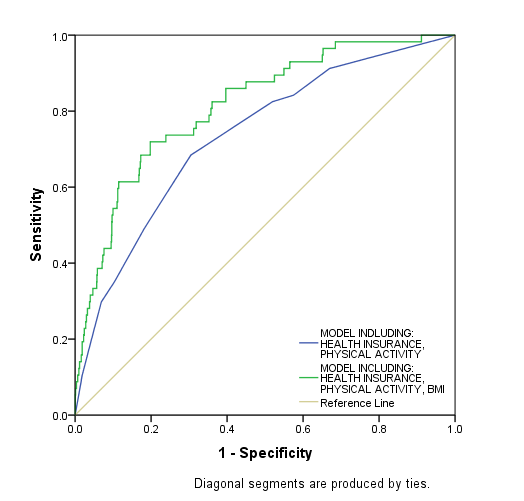

Supplement: Figure S1 — Area under the receiver operating characteristic curves (AUC) for models to discriminate undiagnosed type 2 diabetes compared to no diabetes. The figure shows area under the curves for models to detect undiagnosed type 2 diabetes. The c statistics values were: (1) c: 0.735, (95% CI: 0.668–0.801) for a model including health insurance and physical activity; (2) c: 0.814, (95% CI: 0.758–0.871) for a model including health insurance, physical activity and BMI (continuous). (DOCX) [file pone.0080504.s001.docx]
