# Supplementary material for: The Prevalence and Determinants of Undiagnosed and Diagnosed Type 2 Diabetes in Middle-Aged Irish Adults
Source: PLoS One. 2013 Nov 25;8(11):e80504. doi: 10.1371/journal.pone.0080504 (PMC3840064; doi:10.1371/journal.pone.0080504)
Supplement: Table S1 — Univariate odds ratios (95% CI) of having undiagnosed or diagnosed type 2 diabetes compared to no diabetes. The table displays univariate associations for socio-economic, metabolic and other health related variables with either undiagnosed or diagnosed diabetes. Diagnosed subjects were excluded from models examining undiagnosed diabetes. Undiagnosed subjects were excluded from models examining diagnosed diabetes. (DOCX) [file pone.0080504.s003.docx]

| Table S1. Univariate odds ratios (95% CI) of having undiagnosed or diagnosed type 2 diabetes compared to no diabetes. | | | | | |
| --- | --- | --- | --- | --- | --- |
| Feature | | ***Odds ratio (95% CI) of having undiagnosed T2DM compared to no diabetes^1^*** | | ***Odds ratio (95% CI) of having diagnosed T2DM compared to no diabetes^2^*** | |
|  | | **Odds ratio** | **95% CI** | **Odds ratio** | **95% CI** |
| *Health conditions* | |  |  |  |  |
|  | Male | 1.6 | (1.0-2.6) | 2.3 | (1.5-3.6) |
|  | Age >60 years | 1.3 | (0.8-2.0) | 2.0 | 1.3-3.1) |
|  | On Rx for hypertension | 2.3 | (1.4-3.6) | 5.1 | (3.4-7.8) |
|  | On Rx for cholesterol | 1.9 | (1.2-3.1) | 3.9 | (2.6-5.9) |
|  | **BMI category:** |  |  |  |  |
|  | *<25* | 1 |  | 1 |  |
|  | *25-29.9* | 3.1 | (1.1-8.9) | 11.0 | (2.7-45.6) |
|  | *>30* | 8.7 | (3.1-24.3) | 22.5 | (5.5-92.8) |
|  | Family history of T2DM | 2.0 | (1.2-3.4) | 5.4 | (3.6-8.2) |
|  | CVD | 2.9 | (1.6-5.2) | 4.0 | (2.5-6.3) |
| *Socio-economic* | |  |  |  |  |
|  | **Education:** |  |  |  |  |
|  | *Bachelor or higher* | 1 |  | 1 |  |
|  | *Diploma* | 1.1 | (0.3-4.0) | 0.9 | (0.3-2.9) |
|  | *Secondary* | 1.6 | (0.6-4.5) | 1.6 | (0.6-4.2) |
|  | *Primary only* | 2.6 | (0.9-7.5) | 3.3 | (1.3-8.5) |
|  | **Social class:** |  |  |  |  |
|  | *High income* | 1 |  | 1 |  |
|  | *Middle income* | 1.6 | (0.7-4.0) | 1.4 | (0.7-2.9) |
|  | *Low income* | 1.9 | (0.7-4.8) | 1.5 | (0.8-2.9) |
| *Medical cover* | |  |  |  |  |
|  | **Health insurance:** |  |  |  |  |
|  | *Private insurance* | 1 |  | 1 |  |
|  | *State insurance* | 3.0 | (1.8-5.1) | 2.4 | (1.6-3.6) |
|  | *No insurance* | 3.0 | (1.6-5.6) | 0.8 | (0.3-1.7) |
| *Health behaviours* | |  |  |  |  |
|  | **Physical activity:** |  |  |  |  |
|  | *High* | 1 |  | 1 |  |
|  | *Moderate* | 2.8 | (1.3-6.1) | 1.7 | (1.0-2.7) |
|  | *No physical exercise* | 7.0 | (3.4-14.7) | 1.9 | (1.1-3.3) |
|  | Smoker | 1.2 | (0.8-2.0) | 1.5 | (1.0-2.3) |
|  | **Alcohol use:** |  |  |  |  |
|  | *Non-drinker* | 1 |  | 1 |  |
|  | *Occasional drinker* | 0.7 | (0.4-1.3) | 1. | (0.7-1.8) |
|  | *Regular drinker* | 0.6 | (0.4-1.1) | 0.5 | (0.3-0.8) |
| *Metabolic* | |  |  |  |  |
|  | TAG >1.7 | 3.7 | (2.3-6.0) | 2.0 | (1.3-3.1) |
|  | Non-optimal HDL-C^3^ | 4.9 | (3.0-7.9) | 4.8 | (3.2-7.3) |
|  | Dyslipidaemia^4^ | 7.2 | (4.3-12.2) | 3.9 | (2.3-6.5) |
|  | Hypertension^5^ | 1.6 | (1.0-2.5) | 0.9 | (0.6-1.4) |

^1^Models excluding subjects with diagnosed diabetes. ^2^Models excluding subjects with undiagnosed diabetes.

^3^HDL-C: <1.03 (MALES) <1.29 (FEMALES). ^4^Dyslipidaemia: TAG >1.7 and HDL-C: <1.03 (MALES) <1.29 (FEMALES).

^5^Hypertension: SBP >140 and/or DBP >90.
